# Pro‐inflammatory diet mediates the association between epilepsy and sleep disorders: A cross‐sectional study

**DOI:** 10.1002/ibra.70025

**Published:** 2026-06-18

**Authors:** Juan Yang, Yongsu Zheng, Xuejiao Zhou, Weijin Zheng

**Affiliations:** ^1^ Department of Neurology Affiliated Hospital of Zunyi Medical University Zunyi China; ^2^ Department of Health Management Affiliated Hospital of Zunyi Medical University Zunyi China

**Keywords:** epilepsy, inflammatory diets, mediation analysis, NHANES, sleep disorders

## Abstract

Accumulating epidemiological and mechanistic evidence indicates that anti‐inflammatory dietary patterns may attenuate systematic inflammation underlying chronic disease, including epilepsy (EP), a common and potentially debilitating neurological disorder frequently complicated by sleep disturbances. Herein, we aim to assess the inflammatory potential of habitual diets among individuals with EP, and co‐occurring sleep disorders (SLDs). From 2013 to March 2020, 35,706 participants were enrolled in this study. EP status was defined based on reported use of antiepileptic drugs within the preceding 30 days. SLDs were assessed using questions on habitual sleep duration and validated self‐reported sleep disturbances. Dietary inflammatory index (DII) and energy‐adjusted DII (E‐DII) scores were calculated using 24‐h dietary recall data. Individuals with EP exhibited significantly higher DII and E‐DII scores and a greater prevalence of SLDs compared with individuals without EP. In the fully adjusted model, participants in Quartile 4 of the DII score had significantly increased odds of EP and SLDs, relative to those in Quartile 1. Mediation analyses revealed that DII accounted for 2.650% to 6.234% of the association between EP and SLDs. In stratified analysis, an elevated prevalence of SLDs was observed across Quartile 2–4 for DII and in Quartile 2 and 4 for E‐DII among individuals with EP. Individuals with EP consume diets with greater pro‐inflammatory potential than those without EP. SLDs in this population are associated with higher dietary inflammatory burden—highlighting the need for evidence‐informed, individualized nutritional guidance as part of comprehensive EP management.

## Graphical abstract

This study assesses the inflammatory potential of habitual diets among individuals with epilepsy (EP) and co‐occurring sleep disorders (SLDs). Our findings indicate that SLDs in individuals with EP are associated with a higher dietary inflammatory burden—highlighting the need for evidence‐informed, individualized nutritional guidance as part of comprehensive epilepsy management.

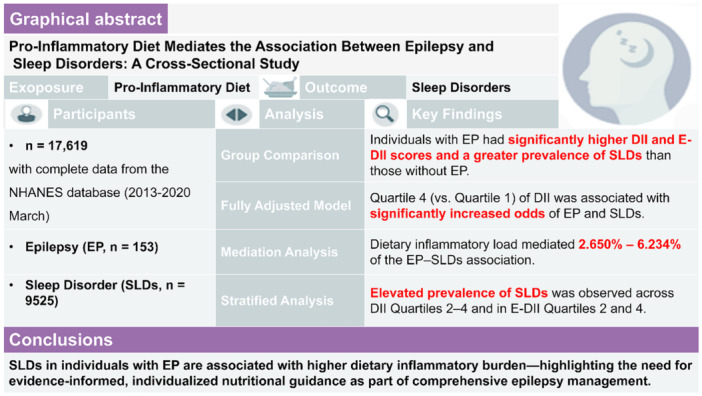

## 1 INTRODUCTION

Dietary intake—particularly natural dietary supplements and plant‐derived adjunctive agents—exerts potential modulatory effects on the pathophysiological mechanisms and integrated management of acute and chronic diseases, including COVID‐19, hypertension, type 2 diabetes, and metabolic syndrome.^1^, ^2^, ^3^, ^4^, ^5^ Inflammation is a key pathophysiological driver of numerous chronic diseases, with dietary patterns playing a well‐established role in modulating systemic inflammatory status.^6^, ^7^, ^8^ Emerging evidence supports the potential of anti‐inflammatory dietary interventions in mitigating inflammation‐associated chronic conditions.^9^

Neuroinflammation, in particular, is recognized as a shared pathological mechanism across a broad spectrum of neurological disorders^10^, ^11^—including epilepsy (EP), a prevalent condition characterized by recurrent, unprovoked seizures resulting from abnormal, hypersynchronous neuronal discharges in the brain. Globally, EP affects over 70 million individuals across all age groups, imposing substantial social, behavioral, health‐related, and socioeconomic burdens.^12^, ^13^ Furthermore, comorbidities—present in 15%–30% of individuals with EP—significantly influence disease trajectory, affecting diagnostic timing, prognosis expectations, therapeutic decision‐making, and treatment outcomes.^14^, ^15^, ^16^ Notably, research indicates that individuals with EP tend to consume diets higher in pro‐inflammatory components.^17^ Additionally, the ketogenic diet, characterized by high fat, low carbohydrate, and adequate protein intake, has demonstrated clinical efficacy in EP management, partly attributable to its anti‐inflammatory properties.^18^

Converging evidence also links pro‐inflammatory dietary patterns with impaired sleep quality.^19^ Sleep disorders (SLDs) are highly prevalent among individuals with EP: while the general adult population exhibits SLD prevalence rates of 10%–18%, these figures rise to 1.4–3.0 times higher among adults with EP.^20^, ^21^ Per the International Classification of Sleep Disorders (ICSD), SLDs encompass insomnia, sleep‐related breathing disorders, central hypersomnolence disorders, circadian rhythm sleep‐wake disorders, parasomnias, sleep‐related movement disorders, and other related conditions.^22^ Critically, co‐occurring SLDs markedly diminish health‐related quality of life in people with EP.^20^ Although bidirectional interactions between sleep disturbances and seizures are well documented—where sleep disturbances can lower seizure thresholds and seizures, in turn, degrade sleep quality—the precise mechanistic interplay between SLDs and EP remains incompletely elucidated.^23^ Preclinical studies using in vitro and in vivo models reveal complex bidirectional interactions among neuroinflammation, epileptogenesis, and sleep dysregulation: seizures trigger inflammatory cascades that may exacerbate sleep dysregulation; conversely, SLDs promote peripheral and central inflammation, and systemic inflammation itself can disrupt sleep homeostasis.^24^, ^25^, ^26^, ^27^ Collectively, these findings point to neuroinflammation as a pivotal biological nexus linking EP and its associated SLDs.

The dietary inflammatory index (DII) is used to assess how dietary exposure contributes to inflammation. Elevated DII scores are associated with pro‐inflammatory diets, whereas lower scores suggest anti‐inflammatory diets.^28^ A cross‐sectional study based on data from the National Health and Nutrition Examination Survey (NHANES) database indicated that the DII was significantly positively correlated with the risk of EP, and this association was statistically presented as a linear dose‐response relationship.^29^ However, current literature lacks robust evidence on whether DII scores independently associate with SLD risk or severity in individuals with EP. We therefore hypothesize that elevated dietary inflammation burden—as indexed by higher DII scores—may contribute to the onset or worsening of SLDs linked to EP.

This study leveraged nationally representative data from the NHANES to investigate the interrelationships among dietary inflammatory potential, EP, and SLDs, with the aim of informing evidence‐based, targeted interventions for the prevention of EP–SLD comorbidity.

## 2 METHODS

### 2.1 Study population

**Figure 1:**
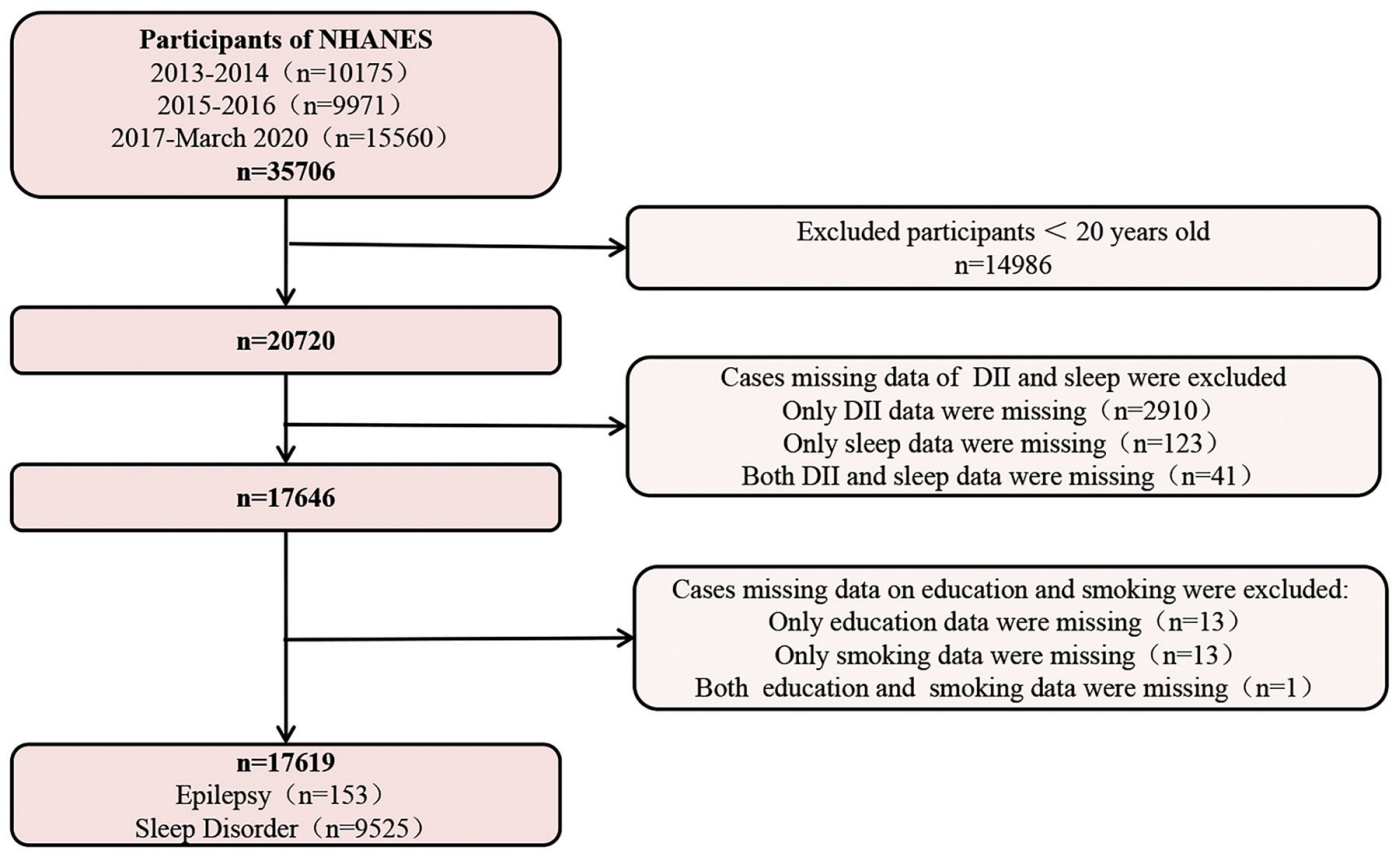
Participant selection flow diagram for the NHANES 2013–2020 cycles.

The participants of this study were recruited from the NHANES, an ongoing cross‐sectional survey that is nationally representative and documents the health and nutritional status of Americans. An in‐depth explanation of the NHANES protocol can be found elsewhere.^30^ The study's data are available on the website (https://www.cdc.gov/nchs/nhanes/). A pooled analysis was conducted on three consecutive NHANES cycles: 2013–2014, 2015–2016, and 2017–March 2020. Among the 35,706 participants initially recruited from 2013 to March 2020, we excluded those younger than 20 years of age (*n* = 14,986). Then, we excluded those lacking DII‐related and sleep‐related data (*n* = 3074). Further, we excluded those lacking data on some covariates, such as education level and smoking status (*n* = 27). The final analysis included 17,619 subjects, comprising 153 individuals with EP and 9525 individuals with SLDs, as shown in Figure 1.

The NHANES study was approved by the NHANES Research Ethics Review Board, and participants gave informed consent in line with the principles of the Helsinki Declaration. Public access to the database was free and not subject to any ethical or administrative restrictions. Additional details can be found at www.cdc.gov/nchs/nhanes/.

### 2.2 Assessment of EP and SLDs

EP and SLDs were ascertained through structured, interviewer‐administered questionnaires during in‐person NHANES examinations. According to the NHANES questionnaire data, participants reported all prescription medications used in the prior 30 days—including drug name, dosage, frequency, and primary indication. Individuals were classified as having EP if they reported current use of ≥1 guideline‐recommended antiseizure medication (e.g., levetiracetam, lamotrigine, valproate) explicitly prescribed for “epilepsy” or “recurrent seizures,” with indication coded under ICD‐10‐CM categories G40 or G40.P.^31^, ^32^

Meanwhile, according to previous studies, in the NHANES surveys conducted from 2013 to March 2020, sleep duration and self‐reported sleep disturbances were assessed using two standardized, validated NHANES items.^33^, ^34^ First, an open‐ended question inquired about habitual sleep duration on weekdays or workdays. Generally speaking, young and older adults should sleep between 6 and 9 h per day, according to the National Sleep Foundation.^35^ Sleep duration was categorized according to the aforementioned guidelines: short duration (≤6 h), normal duration (>6 to <9 h), and long duration (≥9 h). Short or long sleep duration was defined as abnormal sleep duration in this analysis. Second, a dichotomous question—“Have you ever informed a doctor or health professional about your sleep difficulties?”—a validated indicator of perceived sleep impairment requiring medical attention—was used to evaluate self‐reported sleep disturbances. In our study, SLDs were defined as either abnormal sleep duration or self‐reported sleep disturbances.

### 2.3 Assessment of dietary inflammation indicators

The DII was described by Shivappa et al. as a useful tool for assessing dietary inflammation.^36^ Based on a literature review, the DII includes 45 nutrients, foods, and other bioactive dietary compounds correlated with six inflammatory biomarkers: interleukin (IL)‐1β, IL‐4, IL‐6, IL‐10, C‐reactive protein (CRP), and tumor necrosis factor alpha (TNF‐α). In NHANES, participants underwent 24‐h dietary recall interviews to gather comprehensive dietary intake data. According to previous studies, the DII has stable predictive ability when 28 food parameters are used.^37^ The DII scores were calculated using the “Dietaryindex” package in R.^38^ The energy‐adjusted DII (E‐DII) scores were derived by computing DII scores per 1000 calories of dietary intake. In this study, DII and E‐DII scores were analyzed on both categorical and continuous scales. The main analysis was conducted by categorizing DII and E‐DII scores into quartiles. The group exhibiting the highest level of anti‐inflammatory potential (i.e., quartile 1) was designated as the reference category.

### 2.4 Assessments of covariates

Our research utilized the following covariates, as identified in prior studies: age, sex, race/ethnicity, education level, smoking status, family income‐to‐poverty ratio (PIR), body mass index (BMI), and comorbidities. Among these eight covariates, age, PIR, and BMI were treated as continuous variables. Sex and comorbidities were modeled as dichotomous variables, while race/ethnicity, education level, and smoking status were treated as multicategorical variables. The NHANES continuously collected self‐reported race/ethnicity data, categorizing participants into Mexican American, other Hispanic, non‐Hispanic white, non‐Hispanic black, and other/multiracial groups. Education level was classified into five categories: less than 9th grade, 9th–11th grade, high school graduate or general educational development (GED), some college or associate degree, and college graduate or higher. Participants were categorized by smoking status as never smokers, former smokers, and current smokers. PIR was calculated as the ratio of family income to the federal poverty guidelines. BMI was calculated using weight and height measurements obtained at Mobile Examination Centers.

Comorbidities were defined as the presence of one or more of the following conditions: hypertension, coronary heart disease (CHD), heart failure, diabetes, chronic kidney disease, nocturia, stroke, malignancy, or chronic obstructive pulmonary disease (COPD). Information on comorbidities was obtained from self‐reported responses in the personal interview questionnaire and medical history. The definition of nocturia followed that of the Standardization Committee of the International Continence Society.^39^

### 2.5 Statistical analysis

We applied appropriate sampling weights for analysis due to the complex NHANES survey design. Weighted analyses were performed with the R package “survey.” Continuous variables were initially summarized using weighted means (SD), whereas categorical variables were described using weighted frequencies and percentages. Group comparisons for continuous variables were conducted using a t‐test adapted for complex survey samples, and categorical variables were compared using the Rao–Scott chi‐squared test with second‐order correction.

Linear associations between EP or SLDs and the dietary inflammation indicators (DII and E‐DII) were explored using restricted cubic splines (RCSs) with four knots. For DII and E‐DII variables exhibiting non‐linear associations, quartile‐based categorization was applied, with the first quartile serving as the reference group in subsequent analyses.

**Figure 2:**
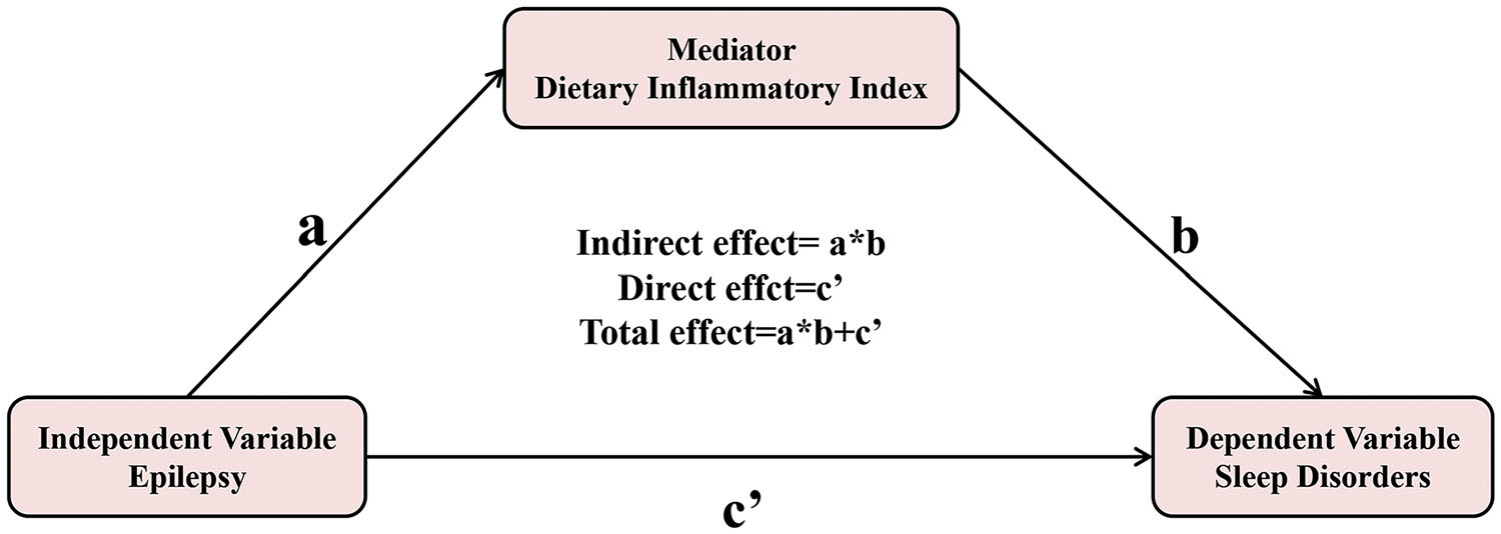
Mediation analysis of the dietary inflammatory index in the association between epilepsy (EP) and sleep disorders (SLDs).

The research examined the association among SLDs, EP, and DII/E‐DII using three multivariate survey‐weighted logistic regression models to account for potential confounders. Model 1 was unadjusted. Model 2 was adjusted for age, sex, and race/ethnicity. Model 3 further adjusted for educational level, smoking status, PIR, BMI, and comorbidities. SLDs were modeled as the dependent variable, and EP was the primary independent variable. We hypothesized that EP may influence SLDs both directly and indirectly—via DII or E‐DII as mediating variables (Figure 2). The magnitude of the mediated effect was quantified as the proportion of the indirect effect relative to the total effect. A statistically significant mediation effect was inferred when DII/E‐DII is significantly associated with both EP and SLDs.^40^ Mediation analyses employed 1000 bootstrap replications to estimate standard errors and confidence intervals. All statistical analyses were conducted in R version 4.4.1 (R Foundation for Statistical Computing, Vienna, Austria). In the current study, a two‐sided *p*‐value < 0.05 was considered statistically significant.

## 3 RESULTS

### 3.1 Baseline characteristics

Our study analyzed 17,619 participants (weighted to 234,007,714 U.S. adults) with complete data from the NHANES database (2013‐2020 March), comprising 48.24% males (*n* = 8496) and 51.76% females (*n* = 9123), with a mean age of 48.09 ± 17.33 years. Table 1 presents the participants' primary baseline characteristics. When stratified by EP, 153 participants (weighted 1,830,020, approximately 0.78%) had EP and 17,466 (weighted to 232,177,694, approximately 99.22%) were EP‐free controls (non‐EP). Education level differed significantly between groups (*p* = 0.02). Compared with controls, the EP group showed a higher proportion of low educational level and a lower proportion of high educational level. Additionally, the EP group had a significantly lower PIR (*p* = 0.03), and significantly higher DII (*p* < 0.001), E‐DII (*p* = 0.002), and prevalence of SLDs (*p* < 0.001). No statistically significant differences were observed between groups in age, sex, race/ethnicity, smoking status, BMI, or comorbidities. When stratified by SLDs, 9525 participants (weighted to 121,778,995, approximately 52.04%) had SLDs, and 8094 (weighted to 112,228,720, approximately 47.96%) were SLD‐free controls (non‐SLDs). Significant differences were observed between groups across multiple factors—including age, sex, race/ethnicity, education level, smoking status, PIR, BMI, comorbidities (all *p* < 0.001). Meanwhile, compared with controls, the SLDs group also showed significantly elevated DII (*p* < 0.001), E‐DII (*p* < 0.001), and prevalence of EP (*p* < 0.001).

**Table 1:** Baseline characteristics of the study participants from the NHANES database from 2013 to 2020.

Variables	Ov erall *N* = 17619 (100%)	Grouped by EP			Grouped by SLDs		
		non‐EP *N* = 17466 (99.22%)	EP *N* = 153 (0.78%)	*p*‐Value	non‐SLDs *N* = 8094 (47.96%)	SLDs *N* = 9525 (52.04%)	*p*‐Value
Age	48.09 (17.33)	48.10 (17.33)	47.21 (17.57)	0.700	46.47 (17.13)	49.59 (17.38)	<0.001
Sex (Male)	8496 (48.24%)	8423 (48.25%)	73 (47.46%)	0.900	4119 (51.79%)	4377 (44.97%)	<0.001
Race				0.600			<0.001
Mexican American	2416 (8.71%)	2399 (8.71%)	17 (8.17%)		1236 (9.56%)	1180 (7.93%)	
Other Hispanic	1887 (6.71%)	1867 (6.71%)	20 (6.62%)		883 (6.75%)	1004 (6.67%)	
Non‐Hispanic White	6657 (63.91%)	6588 (63.87%)	69 (67.97%)		2941 (64.07%)	3716 (63.75%)	
Non‐Hispanic Black	4083 (11.25%)	4050 (11.25%)	33 (11.78%)		1670 (9.45%)	2413 (12.91%)	
Other/Multiracial	2576 (9.42%)	2562 (9.45%)	14 (5.46%)		1364 (10.17%)	1212 (8.73%)	
Education Level				0.020			<0.001
Less Than 9th Grade	1425 (4.09%)	1412 (4.07%)	13 (6.47%)		667 (4.21%)	758 (3.98%)	
9‐11th Grade	2071 (8.38%)	2047 (8.36%)	24 (11.80%)		843 (7.35%)	1,228 (9.34%)	
High School Grad/GED	4069 (24.17%)	4026 (24.08%)	43 (34.69%)		1739 (21.85%)	2330 (26.31%)	
Some College or AA degree	5601 (32.03%)	5554 (32.06%)	47 (28.84%)		2423 (30.32%)	3178 (33.61%)	
College Graduate or above	4453 (31.33%)	4427 (31.43%)	26 (18.20%)		2422 (36.27%)	2031 (26.77%)	
Smoking Status				0.700			<0.001
Never smoker	10,104 (56.92%)	10,030 (56.94%)	74 (53.49%)		5014 (61.68%)	5090 (52.53%)	
Former smoker	4200 (25.25%)	4158 (25.25%)	42 (25.21%)		1796 (24.25%)	2404 (26.18%)	
Current smoker	3315 (17.83%)	3278 (17.80%)	37 (21.30%)		1284 (14.07%)	2031 (21.30%)	
PIR	2.96 (1.61)	2.96 (1.60)	2.38 (1.55)	0.030	3.13 (1.58)	2.80 (1.61)	<0.001
BMI	29.46 (7.02)	29.45 (7.02)	29.98(7.63)	0.500	28.88 (6.53)	29.99 (7.41)	<0.001
Comorbidities	10,531 (55.14%)	10,415 (55.05%)	116 (66.41%)	0.130	4112 (45.41%)	6419 (64.10%)	<0.001
DII	1.02 (2.01)	1.01 (2.01)	1.83 (2.12)	<0.001	0.81 (2.00)	1.21 (2.00)	<0.001
E‐DII	0.91 (1.68)	0.91 (1.68)	1.55 (2.11)	0.002	0.74 (1.56)	1.08 (1.77)	<0.001
EP	153 (0.78%)	—	—	—	39 (0.35%)	114 (1.18%)	<0.001
SLDs	9525 (52.04%)	9411 (51.83%)	114 (78.81%)	<0.001	—	—	—

Abbreviations: AA, associate in arts; BMI, body mass index; DII, dietary inflammatory index; EP, epilepsy; E‐DII, energy‐adjusted DII; GED, general educational development; PIR, income‐to‐poverty ratio; SLDs, sleep disorders.

### 3.2 Curvilinear relationship analysis

**Figure 3:**
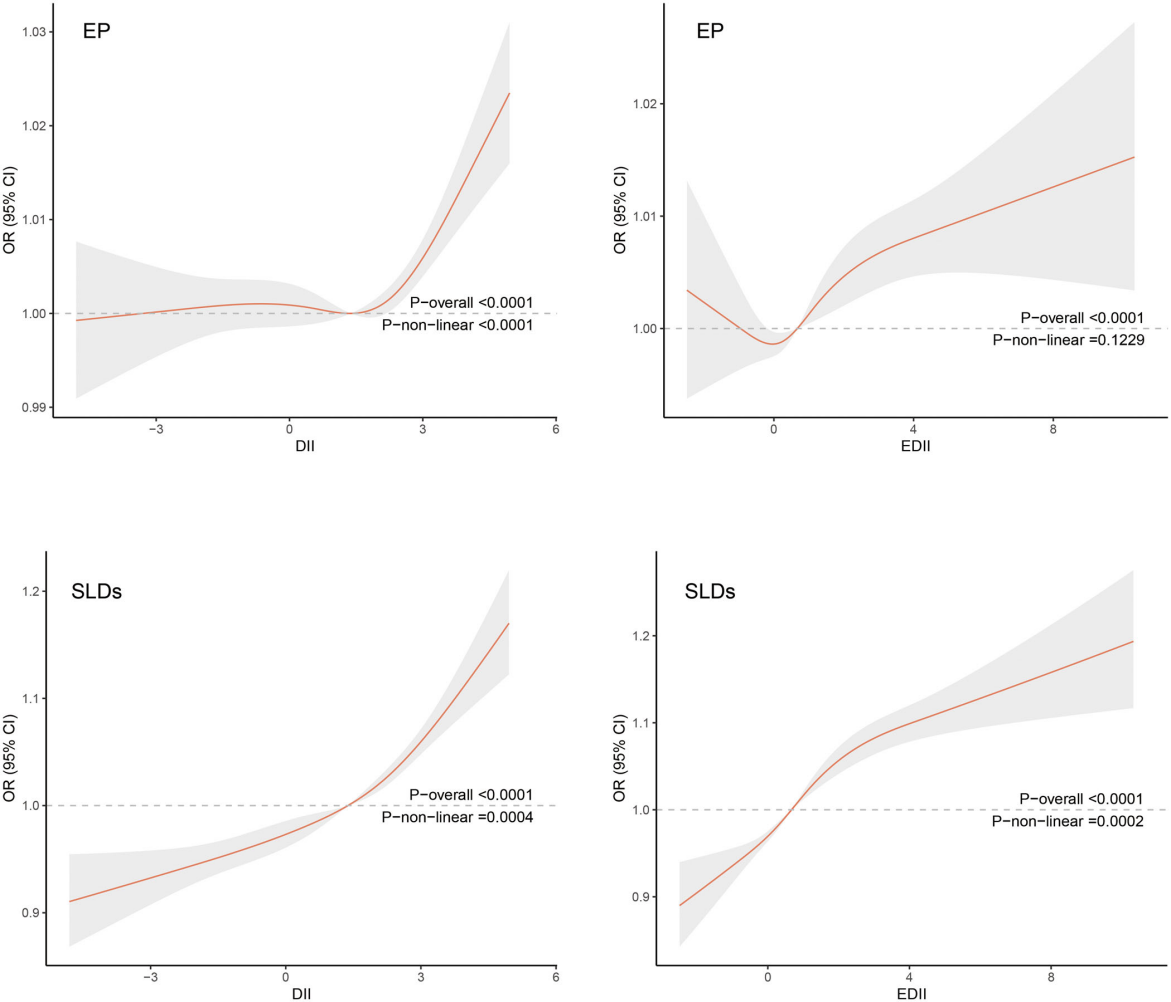
Log‐transformed adjusted ORs and 95% CIs for SLDs and EP associated with the DII and E‐DII scores—Model 3. CI, confidence interval; DII, Dietary Inflammatory Index; E‐DII, Energy‐adjusted DII; EP, epilepsy; OR, odds ratio; SLDs, sleep disorders.

A non‐linear association between EP and DII scores was revealed by RCS (*p* < 0.0001), whereas the association between EP and E‐DII scores was linear (*p* = 0.1229). Additionally, RCS analyses revealed non‐linear associations between SLDs and both DII scores (*p* for non‐linearity = 0.0004) and E‐DII scores (*p* for non‐linearity = 0.0002) (Figure 3).

### 3.3 Relationship among SLDs, EP, and dietary inflammation

All three models demonstrated that participants with EP had a significantly higher risk of SLDs (*p* < 0.001; Model 3: odds ratios [OR] = 3.17, 95% confidence interval [CI]: 1.76–5.68) (Table 2). For the DII score, Quartile 1 was used as a reference. Quartile 4 (most pro‐inflammatory) was significantly associated with higher odds of EP across all models (*p* < 0.01, Model 3: OR = 2.50, 95% CI: 1.28–4.86), whereas Quartiles 2 and 3 showed no significant association (*p* > 0.05). After adjustment for all covariates, a one‐unit increase in the E‐DII score was associated with a higher odds of EP (*p *< 0.01, OR = 1.28, 95% CI: 1.07–1.51) (Table 3).

**Table 2:** Binary logistic regression analysis of the relationship between SLDs and EP.

Models	Model 1	Model 2	Model 3
**EP OR (95% CI)**	3.46 (2.15, 5.57)***	3.54 (2.13, 5.87)***	3.17 (1.76, 5.68)***

*Note*: Model 1 was unadjusted. Model 2 was adjusted for age, sex, and race/ethnicity. Model 3 further adjusted for educational level, smoking status, PIR, BMI, and comorbidities.

Abbreviations: CI, confidence interval; EP, epilepsy; OR, odds ratio; SLDs, sleep disorders.

***p < 0.001.

**Table 3:** Binary logistic regression analysis of the relationship between EP and dietary inflammation scores.

Models	Model 1	Model 2	Model 3
DII score OR (95% CI)			
Quartile 1	Ref	Ref	Ref
Quartile 2	1.36 (0.64, 2.68)	1.37 (0.65, 2.88)	1.24 (0.59, 2.61)
Quartile 3	0.99 (0.44, 2.24)	1.00 (0.45, 2.26)	0.87 (0.38, 1.97)
Quartile 4	3.02 (1.68,5.40)***	3.08 (1.64, 5.82)***	2.50 (1.28, 4.86)**
E‐DII score OR (95% CI)	1.34 (1.17, 1.54)***	1.35 (1.15, 1.58)***	1.28 (1.07, 1.51)**

*Note*: The quartile cut‐points for the DII were as follows: Q1: <:−0.44, Q2: −0.44 to <1.23, Q3: 1.23 to <2.60, Q4: 2.60 to <5.51.

Abbreviations: CI, confidence interval; DII, dietary inflammatory index; EP, epilepsy; E‐DII, energy‐adjusted DII; OR, odds ratio.

**p < 0.01; ***p < 0.001.

Across all models, Quartile 4 of the DII score was significantly associated with a higher risk of SLDs compared to Quartile 1 (*p* < 0.001, Model 3: OR = 1.38, 95% CI: 1.19–1.61). Quartiles 3 and 2 showed similar trends in Models 1 and 2, but these trends were not statistically significant in Model 3. Similarly, across all three models, Quartile 4 of the E‐DII score was significantly associated with increased odds of SLDs (*p* < 0.001; Model 3: OR = 1.34, 95% CI: 1.14–1.57) compared to Quartile 1. The trends for Quartiles 3 and 2 were consistent in Models 1 and 2, but did not reach statistical significance in Model 3 (Table 4).

**Table 4:** Binary logistic regression analysis of the relationship between SLDs and dietary inflammation scores.

Models	Model 1	Model 2	Model 3
DII score OR (95% CI)			
Quartile 1	Ref	Ref	Ref
Quartile 2	1.21 (1.07, 1.37)**	1.18 (1.04, 1.31)*	1.09 (0.96, 1.25)
Quartile 3	1.31 (1.18, 1.46)***	1.25 (1.11, 1.39)***	1.09 (0.98, 1.22)
Quartile 4	1.77 (1.52, 2.06)***	1.66 (1.42, 1.94)***	1.38 (1.19, 1.61)***
E‐DII score OR (95% CI)			
Quartile 1	Ref	Ref	Ref
Quartile 2	1.16 (1.05, 1.29)**	1.16 (1.04, 1.29)**	1.04 (0.93, 1.17)
Quartile 3	1.31 (1.16, 1.49)***	1.26 (1.10, 1.44)***	1.11 (0.97, 1.26)
Quartile 4	1.74 (1.49, 2.02)***	1.60 (1.37, 1.87)***	1.34 (1.14, 1.57)***

*Note*: The quartile cut‐points for the DII were as follows: Q1: < −0.44, Q2: −0.44 to <1.23, Q3: 1.23 to <2.60, Q4: 2.60 to <5.51. The quartile cut‐points for the E‐DII were as follows: Q1: < −0.17, Q2: −0.17 to <0.58, Q3: 0.58 to <1.56, Q4: 1.56 to <10.63.

Abbreviations: CI, confidence interval; DII, dietary inflammatory index; E‐DII, energy‐adjusted DII; OR, odds ratio; SLDs, sleep disorders.

*p < 0.05; **p < 0.01; ***p < 0.001.

### 3.4 Mediation analysis

A mediation analysis was conducted to examine whether DII and E‐DII scores statistically mediate the association between EP and SLDs. Results indicated statistically significant indirect effects for both the DII and E‐DII scores across all models (all *p* < 0.01). In Model 3, the DII score accounted for 2.650% of the total effect of EP on SLDs, with an indirect effect estimate of 0.00644. Similarly, in Model 3, the E‐DII score accounted for 3.487% of the total effect of EP on SLDs, with an indirect effect estimate of 0.00859 (Table 5).

**Table 5:** Mediation effect of dietary inflammation scores between EP and SLDs.

Mediator	Direct effect	Indirect effect	Total effect	Proportion mediated (%)
DII score				
Model 1	0.25129***	0.01695***	0.26824***	6.234***
Model 2	0.25294***	0.01419***	0.26713***	5.245***
Model 3	0.23064***	0.00644**	0.23708***	2.650**
E‐DII score				
Model 1	0.25269***	0.01611**	0.26880***	5.924**
Model 2	0.25285***	0.01315***	0.26599***	4.842***
Model 3	0.23364***	0.00859**	0.24223***	3.487**

**p < 0.01; ***p < 0.001.

Abbreviations: DII, dietary inflammatory index; E‐DII, energy‐adjusted DII; EP, epilepsy; SLDs, sleep disorders.

### 3.5 Stratified analysis

In the stratified and interaction analyses, DII and E‐DII scores were categorized into quartiles, and results were adjusted using a fully adjusted model. In Quartile 1 of both DII and E‐DII scores, stratified analysis revealed no significant association between EP and SLDs (*p* > 0.05). In Quartile 2, both DII (*p* < 0.05; OR = 3.71, 95% CI: 1.31–10.50) and E‐DII (*p* < 0.01; OR = 4.03, 95% CI: 1.49–10.90) scores showed significant associations with EP regarding SLDs. In Quartile 3, the DII score was significantly associated with EP and SLDs (*p* < 0.01; OR = 5.66, 95% CI: 1.68–19.10), whereas the E‐DII score showed no significant association (*p* > 0.05; OR = 2.27, 95% CI: 0.78–6.63). In Quartile 4, both DII (*p* < 0.05; OR = 5.01, 95% CI: 1.30–19.40) and E‐DII (*p* < 0.01; OR = 8.81, 95% CI: 2.05–37.80) scores demonstrated significant associations with EP regarding SLDs. Interaction analysis revealed no statistically significant interaction between EP and either DII or E‐DII scores (Table 6).

**Table 6:** Association between epilepsy and SLDs stratified by categories of dietary inflammation scores.

Mediator	Model 4 OR (95% CI)	*p* for interaction
DII score		
Quartile 1	1.00 (0.30, 3.29)	0.2
Quartile 2	3.71 (1.31, 10.50)*	
Quartile 3	5.66 (1.68, 19.10)**	
Quartile 4	5.01 (1.30, 19.40)*	
E‐DII score		
Quartile 1	1.00 (0.31, 3.27)	0.094
Quartile 2	4.03 (1.49, 10.90)**	
Quartile 3	2.27 (0.78, 6.63)	
Quartile 4	8.81 (2.05, 37.80)**	

*Note*: The quartile cut‐points for the DII were as follows: Q1: < −0.44, Q2: −0.44 to <1.23, Q3: 1.23 to <2.60, Q4: 2.60 to <5.51. The quartile cut‐points for the E‐DII were as follows: Q1: < −0.17, Q2: −0.17 to <0.58, Q3: 0.58 to <1.56, Q4: 1.56 to <10.63.

Abbreviations: CI, confidence interval; DII, dietary inflammatory index; E‐DII, energy‐adjusted DII; OR, odds ratio; SLDs, sleep disorders.

*p < 0.05; **p < 0.01.

## 4 DISCUSSION

This study bridges critical gaps in the current literature: while etiology, pathophysiology, and therapeutics of EP and SLDs have been extensively investigated, their bidirectional clinical and mechanistic interplay—and particularly the role of modifiable lifestyle factors such as diet—remains incompletely characterized. Accumulating evidence indicates that dietary patterns significantly influence chronic disease risk; for instance, anti‐inflammatory dietary patterns—including the Dietary Approaches to Stop Hypertension, Mediterranean, and “prudent” diets—are consistently associated with reduced incidence and severity of multiple chronic conditions.^41^, ^42^ Conversely, pro‐inflammatory dietary patterns, quantified by validated indices such as the DII and its E‐DII, have emerged as potential predictors of systemic inflammation and related morbidities.^43^ Notably, Inflammation is increasingly recognized as a shared pathological substrate linking both epileptogenesis and SLD pathogenesis, while the relationship among inflammatory dietary patterns, EP and SLDs has received scant empirical attention. Leveraging nationally representative data from NHANES (2013–2020), we found that U.S. adults with EP exhibited significantly higher DII and E‐DII scores—indicating greater habitual intake of pro‐inflammatory foods and nutrients—compared with those without EP. Similarly, individuals with SLDs demonstrated elevated dietary inflammatory burden. Most importantly, mediation analyses revealed that heightened dietary inflammatory potential partially explains the association between EP and concurrent SLDs, suggesting neuroinflammation may serve as a plausible biological pathway underlying this comorbidity.

Our findings confirm that individuals with EP exhibit significantly higher DII and E‐DII scores relative to EP‐free controls (non‐EP)—consistent with prior evidence demonstrating a pro‐inflammatory dietary pattern in this population. Recent studies report that, compared with healthy controls, individuals with EP consume fewer anti‐inflammatory foods and nutrients—including fruits, vegetables, and micronutrients such as vitamins C, D, and E—while exhibiting elevated intake of pro‐inflammatory items, particularly sugar‐sweetened beverages and saturated fats.^29^, ^44^ Similarly, Fernandez et al. documented higher intakes of refined carbohydrates and total protein, alongside lower consumption of anti‐inflammatory fatty acids—specifically monounsaturated (MUFA) and polyunsaturated fatty acids (PUFA)—among persons with EP.^45^ Collectively, these convergent observations reinforce the robustness of our primary finding: EP is associated with a quantifiably greater habitual exposure to dietary pro‐inflammatory components, as objectively captured by validated, population‐based inflammatory indices.

Individuals with EP frequently experience comorbid SLDs, and accumulating evidence suggests that dietary inflammatory potential is a shared, modifiable pathophysiological link underlying both conditions. Our findings demonstrate a statistically significant association between elevated dietary inflammatory burden—as quantified by the DII—and increased prevalence of SLDs, corroborating prior epidemiological observations.^19^ Consistent with this, individuals with SLDs exhibited significantly higher DII scores compared with SLD‐free (non‐SLDs) controls, aligning with previous reports linking pro‐inflammatory dietary patterns to sleep disruption. Importantly, bidirectional pathways are plausible: while SLDs may promote unhealthy lifestyle behaviors—including increased intake of ultra‐processed, high‐sugar, and high‐saturated‐fat foods—dietary inflammation itself may exacerbate sleep dysregulation through neuroinflammatory and autonomic mechanisms. Therefore, integrating evidence‐based dietary interventions into clinical management represents a promising strategy for both preventing and mitigating SLDs in at‐risk populations.

Given the substantial clinical risks associated with EP and SLDs, considerable research efforts have focused on elucidating their etiological mechanisms and identifying modifiable preventive targets. A study revealed that neuroinflammation may serve as a key pathophysiological bridge linking EP and SLDs.^46^ Furthermore, multiple independent studies have demonstrated that dietary patterns and specific nutrients exert potent regulatory effects on neuroinflammatory processes.^47^, ^48^, ^49^ However, to date, the role of dietary inflammatory potential in individuals with EP and co‐occurring SLDs has not been investigated. In our study, both the mean DII score and the prevalence of SLDs were significantly higher in individuals with EP than in EP‐free controls (non‐EP). We conducted formal mediation analysis to examine the mediating effect of the DII score on the association between EP and SLDs. Our findings indicate that the proportion of the total effect mediated by DII ranged from 2.650% to 6.234% across multiple adjustment models, suggesting that dietary inflammatory potential may contribute to SLD development in individuals with EP. Consistent with our hypothesis, stratified analysis revealed no increased SLD prevalence among individuals with EP with low dietary inflammation. In contrast, those with moderate dietary inflammation exhibited a significantly higher incidence of SLDs, supporting a dose–response–like relationship. Collectively, these findings imply that a chronically pro‐inflammatory diet may facilitate or exacerbate SLD onset in individuals with EP.

## 5 CONCLUSION

This study is the first to investigate dietary inflammation among individuals with EP with SLDs, yielding findings with potential implications for clinical practice and public health interventions. Nevertheless, several limitations warrant careful consideration. First, the cross‐sectional design precludes causal inference and limits control over potential confounding factors. For instance, EP was defined solely on the basis of self‐reported use of anti‐epileptic drugs within the preceding 30 days—a criterion that may both underidentify individuals with untreated or well‐controlled EP and misclassify individuals using these medications for non‐epileptic indications (e.g., neuropathic pain or migraine prophylaxis). Similarly, SLDs were identified via self‐reported sleep duration abnormalities or subjective sleep disturbances, which—while pragmatic in large‐scale surveys—lack diagnostic specificity and may introduce heterogeneity and misclassification bias. However, standardized sleep assessments (e.g., validated questionnaires or polysomnography‐based criteria) were not available in the NHANES dataset. Second, adiposity was adjusted for using BMI alone, a measure that does not distinguish between fat mass and lean body mass and thus may inadequately account for adiposity‐associated inflammatory pathways. This constraint arises from the unavailability of objective body composition metrics—such as those derived from bioelectrical impedance analysis or dual‐energy X‐ray absorptiometry—in all NHANES survey cycles. Third, objective physical activity data (e.g., accelerometer‐derived metrics) were not included in our NHANES sample, despite robust evidence linking physical activity to systemic inflammation, sleep quality, and EP outcomes. Fourth, dietary inflammation scores were computed using only 62.2% (28 out of 45) of the original food parameters due to item‐level missingness in dietary recall data. Collectively, these methodological constraints should inform the interpretation of our results and guide the design of future prospective and mechanistically oriented studies.

Our study indicates that individuals with EP are more likely to consume foods with high inflammatory potential, thereby increasing their risk of SLDs. Previous studies have found that DII exerts a modest mediating effect (ranging from 2.31% to 12.25%) in EP— related moderate to severe depression (MSD).^17^ Similarly, our mediation analysis suggests that dietary inflammation may contribute to SLD development in individuals with EP, albeit with a relatively small mediated proportion (ranging from 2.650% to 6.234%). These suggest that the DII may reflect a broader role for dietary inflammatory load in the occurrence of EP‐related comorbidities. In conclusion, EP may exert both direct and indirect effects on SLDs. Increasing consumption of anti‐inflammatory foods and nutrients may help prevent SLDs in individuals with EP. Additional prospective investigations are required to confirm this association and clarify whether diet acts as a moderator or mediator.

## AUTHOR CONTRIBUTIONS

Juan Yang contributed to the research strategy design, data extraction and verification, and manuscript drafting. Yongsu Zheng contributed to data management, statistical analysis, and drafting of the manuscript. Xuejiao Zhou contributed to creating figures and tables and drafting the manuscript. Weijin Zheng contributed to the research strategy design, research supervision, data verification, and manuscript revision. All authors participated in the manuscript preparation and approved the final submitted version.

## CONFLICT OF INTEREST STATEMENT

The authors declare no conflicts of interest.

## ETHICS STATEMENT

This study used de‐identified data from the NHANES study. The original NHANES study was approved by the NHANES Research Ethics Review Board, and participants gave informed consent in line with the principles of the Helsinki Declaration. As this research constitutes a secondary analysis of publicly available anonymized data, it was exempt from requiring additional ethical approval.

## Data Availability

The data that support the findings of this study are openly available in NHANES at https://www.cdc.gov/nchs/nhanes/index.html.
